# Steady Boundary Layer Slip Flow along with Heat and Mass Transfer over a Flat Porous Plate Embedded in a Porous Medium

**DOI:** 10.1371/journal.pone.0114544

**Published:** 2014-12-22

**Authors:** Asim Aziz, J. I. Siddique, Taha Aziz

**Affiliations:** 1 College of Electrical and Mechanical Engineering, National University of Sciences and Technology, Rawalpindi, 46000, Pakistan; 2 Department of Mathematics, Pennsylvania State University, York Campus, 1031 Edgecomb Avenue, York, Pennsylvania, 17403, United States of America; 3 School of Computational and Applied Mathematics, DST-NRF Centre of Excellence in Mathematical and Statistical Sciences, Differential Equations, Continuum Mechanics and Applications, University of the Witwatersrand, Wits, South Africa; 4 School of Natural Sciences, National University of Sciences and Technology, Sector H-12, Islamabad, Pakistan; University of Zurich, Switzerland

## Abstract

In this paper, a simplified model of an incompressible fluid flow along with heat and mass transfer past a porous flat plate embedded in a Darcy type porous medium is investigated. The velocity, thermal and mass slip conditions are utilized that has not been discussed in the literature before. The similarity transformations are used to transform the governing partial differential equations (PDEs) into a nonlinear ordinary differential equations (ODEs). The resulting system of ODEs is then reduced to a system of first order differential equations which was solved numerically by using Matlab bvp4c code. The effects of permeability, suction/injection parameter, velocity parameter and slip parameter on the structure of velocity, temperature and mass transfer rates are examined with the aid of several graphs. Moreover, observations based on Schmidt number and Soret number are also presented. The result shows, the increase in permeability of the porous medium increase the velocity and decrease the temperature profile. This happens due to a decrease in drag of the fluid flow. In the case of heat transfer, the increase in permeability and slip parameter causes an increase in heat transfer. However for the case of increase in thermal slip parameter there is a decrease in heat transfer. An increase in the mass slip parameter causes a decrease in the concentration field. The suction and injection parameter has similar effect on concentration profile as for the case of velocity profile.

## Introduction

In recent years the theory of boundary layer has become important due to its application in modern engineering innovations and industrial processes. One of the most common application of the boundary layer theory is calculation of the friction drag of bodies in the flow, for example, the drag of a flat plate at zero incidence, the friction drag of a ship, an airfoil, the body of an airplane, or a turbine blade etc. Prandtl [1] introduced boundary layer theory to understand the flow structure of a viscous fluid near a solid boundary. The early contribution in this field is due to Blasius [2]. Blasius solved the famous boundary layer equation for a flat moving plate problem and found a power series solution of the model. Falkner and Skan [3] generalized the Blasius problem by considering the boundary layer flow over a wedge inclined at a certain angle. A numerical solution of the classical Blasius problem was presented by [4]. Sakiadis [5] investigated the boundary layer flow over a continuously moving rigid surface with a constant speed. Cortell [6] provided the numerical solution of Sakiadis flow by including the radiation effects on the boundary layer. Crane [7] was the first who investigated the boundary layer flow due to a stretching surface and find the exact solutions of the boundary layer equations. A detailed literature review on boundary layer theory and related topics is given by [8]. Problems involving convective boundary conditions for the Blasius flow have been investigated by [9]–[11]. Recently, the authors in [12], [13] analyzed boundary layer flow and heat transfer of non-Newtonian fluid models over a stretching surface.

Boundary layer flows and heat and mass transfer characteristics of a fluid in a porous media have been studied extensively because such processes exist in nature and have many engineering applications. Examples include heat exchanger, recovery of petroleum resources, fault zones, catalytic reactors, cooling devices, chemical reaction in a reactor chamber consisting of rectangular ducts, deposition of chemical vapor on surfaces and so on. Gebhart and Pera [14] showed the nature of vertical natural convection flows resulting from the combined buoyancy effects of thermal and mass diffusion. Heat and mass transfer on a stretching surface with suction and blowing was investigated by [15]. The effect of mass transfer on free convective hydromagnetic oscillatory flow past an infinite vertical porous plate is estimated by [16]. Khaled and Vafai [17], discussed various flow models in porous medium with applications in biological areas such as diffusion in brain tissues, tissue generation process, blood flow in tumors, bio-heat transfer in tissues and bio-convection. A comprehensive literature survey on these studies can be found in the books of [18]–[20].

Non-Darcian effects on a vertical plate and natural convection in porous media was studied by [21]. Kaviany [22], [23] used the Darcy–Brinkman model to study the effects of the presence of boundary and inertia forces on the natural or forced convection heat transfer rate over a fixed impermeable heated plate embedded in a porous medium. Non-Darcian effects on forced convection heat transfer over a flat plate in a highly porous medium was investigated by [24]. Alazmi and Vafai [25] in their work provided an analysis of the free surface fluid flow and heat transfer through porous media. A computational analysis of heat transfer is performed by [26] for the forced convection fluid flow on a heated flat plate embedded in a porous medium. [27] found that, there is a temperature slip due to a convective boundary condition at the wall. The temperature slip is mainly affected by the mass transfer parameter, the Prandtl number and the parameters associated with the stretching/shrinking of the bounding surface. The aim of [28], is to present an exact analysis of combined effects of radiation and chemical reaction on the magnetohydrodynamic (MHD) free convection flow of an electrically conducting incompressible viscous fluid over an inclined plate embedded in a porous medium. Recently, [29] in their work studied the effects of an arbitrary wall shear stress on unsteady magnetohydrodynamic (MHD) flow in a porous medium with conjugate effects of heat and mass transfer.

In aforementioned investigations all the authors utilized the no-slip condition at the boundary. There are many physical situations where no-slip conditions are not valid (see for example, [30]–[32]). These studies provide a reasonable justification to apply the partial slip boundary condition relating to the shear rate at the boundary. Beavers and Joseph [30] first used the partial slip condition for the flow past the permeable wall. The effects of slip boundary condition for fluid flow over a stretching sheet are discussed by [31], [32]. Martin and Boyd [33] considered slip boundary condition for the heat and mass transfer in a laminar boundary layer flow over a flat plate. In [34] the authors presented an unsteady magnetohydrodynamic convective heat and mass transfer past a vertical permeable plate using slip boundary conditions with thermal radiation and chemical reaction. Continuing on similar lines, Bhattacharyya et al. in [35] employed velocity and thermal slip boundary conditions on a forced convective boundary layer flow and heat transfer of an incompressible fluid past a porous plate embedded in a porous medium. Another interesting study that incorporates the slip conditions on a magnetohydrodynamic (MHD) slip flow of ferro fluid along the stretching cylinder is presented by [36]. In [37], [38] authors presented the analytical solutions to indicate the implications of introducing a thin gas layer on the hydrodynamic aspects of the two phase gas-power law liquid. Recently, [39] in their work study the flow and heat transfer of carbon nanotubes (CNTs) along a flat plate subjected to Navier slip and uniform heat flux boundary conditions.

In this paper, a numerical study is carried out on simplified model of an incompressible fluid flow with heat and mass transfer over a flat porous plate sandwiched in a porous medium. Partial and thermal slip boundary conditions are employed as discussed by [35]. In addition to the previous work of Krishnendu, we include mass slip condition at the boundary which gives interesting features related to engineering applications for example, reverse osmosis filters. Similarity approach is employed to transform the governing system of partial differential equations to a system of ordinary differential equations togather with the boundary conditions. The resulting ODE system is solved numerically. The results presented in this paper with the desire to expand the scope of previously done work of [35].

## Mathematical Model

**Figure 1:**
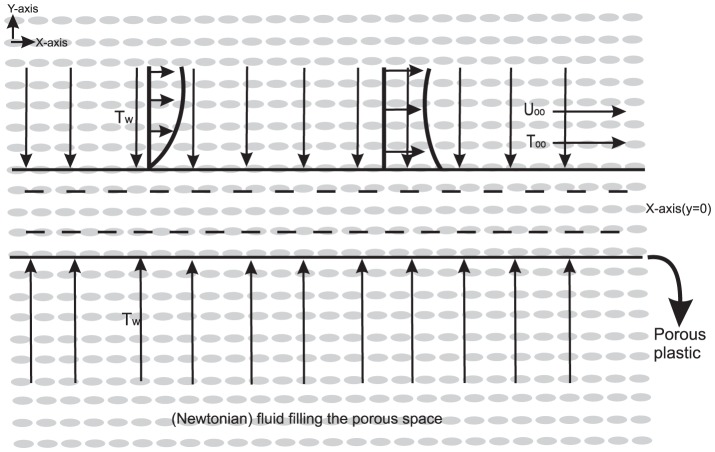
Schematic representation of geometry.



Consider steady two dimensional flow of an incompressible viscous fluid with heat and mass transfer over a porous flat plate embedded in a porous medium. The *x*-axis is taken along the plate and the *y*-axis is taken normal to the plate. The geometry of the flow model is given in Fig. 1. In order to simplify the model the following assumptions are made: the fluid flow is taken as laminar and stable, all body forces are neglected and all properties are assumed independent of temperature. In view of the above assumptions, as well as of the usual boundary layer approximations the following system of PDEs for the flow along with heat and mass transfer is obtained.

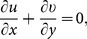
(1)

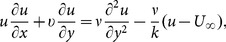
(2)

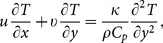
(3)

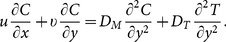
(4)


In the above system of equations, 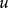 and 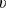 represent velocity in 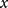 and 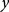 directions respectively, 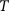 is the fluid temperature, and 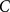 is the mass concentration. The 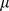 represent the fluid viscosity, 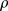 the fluid density and the ratio 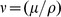 is the kinematic viscosity. The constant parameters in the system are: 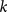 the permeability of porous material, 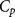 the specific heat at constant pressure, 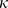 the thermal conductivity of the fluid, 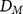 the molecular diffusivity and 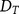 the thermal diffusivity.

The appropriate partial slip boundary conditions for the velocity, temperature and concentration boundary conditions are given by
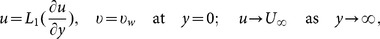
(5)

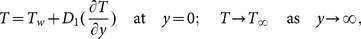
(6)and
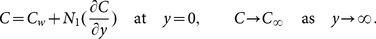
(7)


In equations (5)–(7) 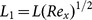 is the velocity slip factor, 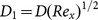 is the thermal slip factor and 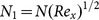 mass slip factor. Here 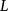, 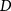 and 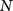 are the initial values of velocity, thermal and mass slip factors respectively. The 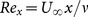 is the local Reynolds number and 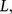 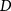 and 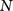 all three have dimensions of length. Moreover, 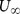 is the the free stream velocity, 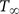 is the free stream temperature and 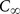 is the free stream mass concentration. In these equations 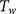 and 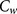 represent the temperature of the plate and the mass concentration. The velocity 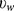 defines suction or blowing through the porous plate and is written as 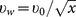. In this relation 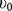 is constant linked with suction if 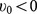 and blowing when 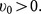


## Solution of the Problem

In this work similarity technique is used to solve the system of equations (1)–(4) along with the boundary conditions (5)–(7). The similarity transformations are [35]

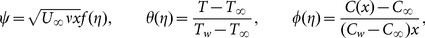
(8)where 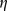 is similarity variable defined as 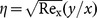.

Introducing the above transformations in equations (1)–(4), we obtain the self-similar system of ordinary differential equations
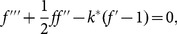
(9)

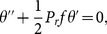
(10)

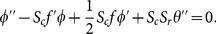
(11)


In equation (9), 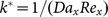 represents the permeability of porous medium and 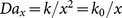 is the local Darcy number. Here 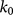 is the constant. In equation (10) 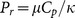 is the Prandtl number. Finally in equation (11), 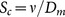 is the Schmidt number and 
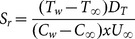
 is the Soret number.

The boundary conditions (5)–(7) transforms to the following form:
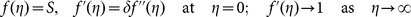
(12)and
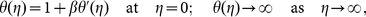
(13)

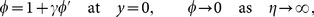
(14)where 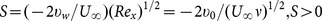 (i.e. 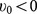) corresponds to suction and 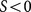 (i.e. 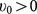) corresponds to blowing, 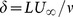 is the velocity slip parameter, 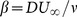 is the thermal slip parameter and 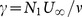 is a mass slip parameter.

The nonlinear coupled ordinary differential equations (9)–(11) subject to boundary conditions (12)–(14) are solved numerically using Matlab bvp4c solver. In order to use bvp4c, first we have to convert (9)–(11) to a system of first order differential equations
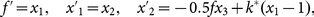
(15)

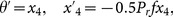
(16)

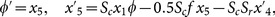
(17)and boundary conditions becomes
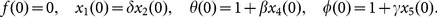
(18)


Note that bvp4c uses a collocation method and requires an initial guess for the desired solution for the ordinary differential equations (9)–(11). In order to make an appropriate guess we start with a set of parameter values for which solution was known and progress until we obtain the solution of our problem. In this case the obtained solution was in good agreement with the previous studies of [40] and [35] for 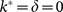.

In the section below results are summarized on the basis of different values of parameters such as non-dimensional permeability 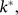 velocity 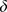, thermal 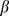, and mass concentration 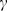 slip parameters. In addition to these we have also explored the influence of Prandtl number 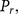 Smith number 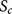 and Soret number 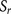 on velocity, temperature and mass transfer profiles.

## Results and Discussion

**Figure 2:**
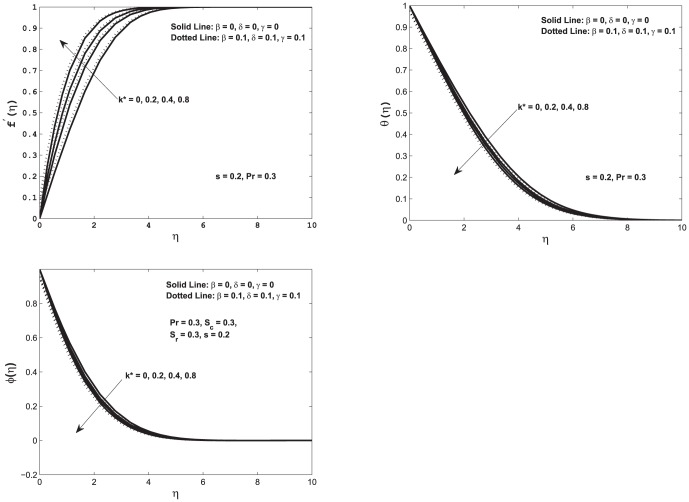
Top left: Velocity profile 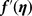 as a function of 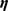; Top right: Temperature profiles 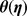 as function of 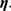 Bottom left: Concentration profile 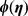 as function of 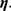 In all of these figures we vary permeability 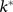 with slip and no–slip conditions.

**Figure 3:**
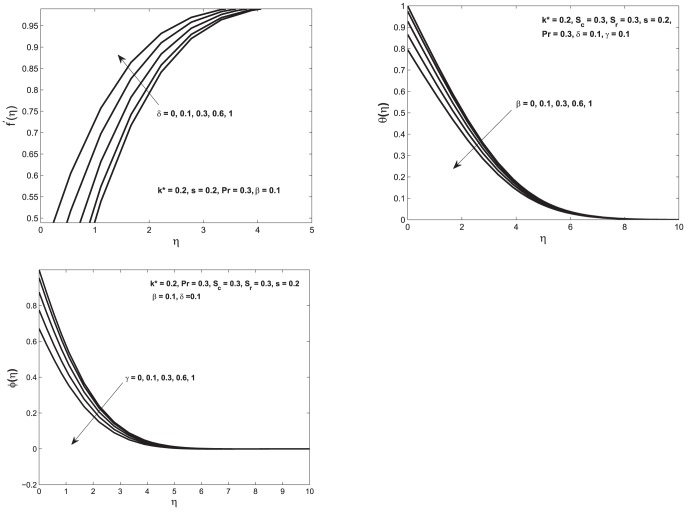
Top left: Velocity profile 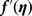 as a function of 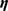; Top right: Temperature profiles 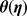 as function of 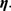 Bottom left: Concentration profile 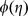 as function of 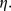 In all of these figures we vary velocity, thermal and concentration slip parameters for both slip and no–slip conditions.

**Figure 4:**
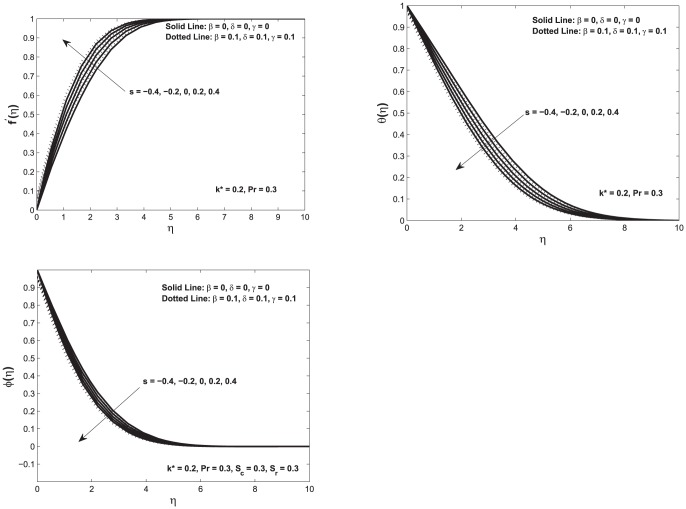
Top left: Velocity profile 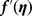 as a function of 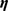; Top right: Temperature profiles 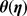 as function of 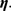 Bottom left: Concentration profile 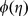 as function of 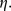 Here we vary the suction/blowing parameters for both slip and no–slip conditions.

**Figure 5:**
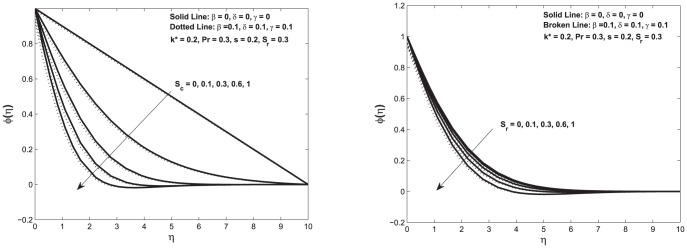
Left: Concentration profile 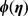 as a function of 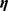; Right: Concentration profile 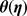 as function of 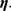 Here we vary Schmidt and Soret numbers for both slip and no-slip conditions.

In order to examine and understand the behavior of various physical parameters of the flow problem, Figs. 2–5 have been plotted. Fig. 2 shows the effect of the permeability 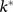 on velocity, temperature, and concentration profiles with slip and no-slip conditions at the boundary. It is observed the velocity along the plate increases which in turn decreases thickness of the momentum boundary layer for both slip and no-slip conditions. An increase in the porosity of the medium decreases the magnitude of the Darcian body force which decelerates the particles in the fluid. The variation in 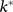 enhances the motion of the fluid in the boundary layer. Influence of mass concentration equation in velocity profile in both slip and no-slip case is not noticeable. In summary we conclude that the increase in permeability of porous medium, decrease in Darcian body force occurs which ultimately decelerates the fluid particles in the porous medium. In top right figure, when permeability of the porous medium is increased for slip and no-slip cases, the temperature 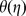 decreases at a point and decreases the thermal boundary layer thickness. In other words this increase of permeability of porous medium decreases the thickness of momentum boundary layer which eventually increases the heat transfer. It is important to notice that similar trend was observed when mass transfer was not present (see [35]). Bottom left figure shows the concentration profile as a function of 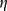 for different values of permeability parameter 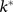. Here we observe that the concentration profile decreases when permeability is increased. When the permeability of the porous medium is increased the porous medium becomes more porous which causes the decrease in magnitude of the Darcian body force and the rate of mass transfer into the porous medium is increased. Fig. 3 shows the effect of variation in velocity, temperature and concentration profile as we vary the velocity, thermal and concentration slip parameters. The increase in velocity slip parameter 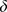 allows the increase in fluid flow past the plate and decrease in the thickness of boundary layer as shown in top left figure in Fig. 3. The increase in thermal slip parameter 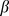 decrease the temperature. The reason for this decrease in temperature is based on the fact that amount of heat transferred from the plate to the fluid is decreased and causes to decrease the temperature as shown in top right figure in Fig. 3. It is important to notice that the influence of thermal slip on velocity profile is not noticed as momentum equation is not coupled with 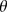. Both, velocity and temperature profiles observations are consistent with [35] observations. The increase in concentration slip parameter shows the decrease in concentration profile. This decrease in concentration profile is due to an increase in the mass transfer from the fluid to the porous medium as shown in bottom left figure. Fig. 4 shows the variation in velocity, temperature and concentration profile respectively as a function of 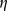 for various values of suction parameter. Herein, 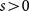 shows the suction and 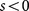 shows the blowing. For 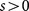; fluid velocity increases as the fluid particles are sucked in the porous wall, which in turn reduces both the fluid boundary layer and the thickness of momentum boundary layer. In short, we can summarize that due the suction behavior, the velocity increases and the fluid and thickness of momentum boundary layer decreases as can be seen in top left Fig. 4. On the other hand, for the case of blowing i.e. 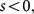 the opposite trend is observed. The effect in temperature profile due to suction and blowing is shown in top right Fig. 4. When suction is increased 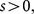 that refers to bringing the fluid close to the wall, this causes a decrease in temperature profile and also decreases the thermal boundary layer. This entire phenomenon causes an increase in the rate of heat transfer. An opposite trend can be seen for the case of blowing 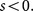 In addition to these observation bottom left figure shows the variation in concentration profile for the case of suction 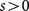 and blowing 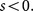 The increase in suction 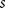 parameter causes a decrease in the concentration field. An opposite trend can be seen for the case of blowing 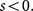 Fig. 5 shows the variation in concentration profile due to the change in Schmidt and Soret numbers.

It is observed that the increase in Schmidt number decreases the concentration profile. This decrease in concentration profile is slow and steady for both slip and no-slip cases as seen in top left Fig. 5. We observe the similar trend due to the change in Soret numbers. The decrease in concentration causes the increase in the mass transfer from the fluid to the porous medium as seen in top right Fig. 5.

## Concluding Remarks and Future Work

In this paper forced convective boundary layer flow along with heat and mass transfer over a porous plate in a porous medium is presented. The reduced coupled system of ODEs was solved numerically. Our observations have been based on permeability, slip, no slip and suction parameters. We have also summarized observations based on Schmidt number and Soret number. In the end we present the future directions.

When the permeability of the porous medium is increased, the velocity of the fluid increases and temperature profile decrease. This happens due to a decrease in drag of the fluid flow. When the velocity slip parameter is increased the velocity of fluid increases whereas temperature profile shows a decrease as shown in [35]. When the thermal slip is increased a temperature profile decreases at a point.

In the case of heat transfer, the increase in permeability and slip parameter causes an increase in heat transfer but for the case of increase in thermal slip parameter causes a decrease in heat transfer. For the case of Schmidt number 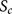 and Soret number 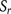, the concentration profile decrease for both slip and no–slip cases. An increase in the mass slip parameter causes a decrease in the concentration field. The suction and injection parameter has similar effect on concentration profile as for the case of velocity profile. The presented study is a simple but an important extension of work presented by [35], by including a mass transfer in the system. The inclusion of mass transfers in their study expands the scope of their effort in many other engineering and scientific fields.

The present model has exploited a number of simplifications in order to focus on the principal effects of heat and mass transfer, permeability, slip and no-slip parameters. An interesting area to explore in future investigations would be the use of temperature dependent thermal conductivity and heat flux boundary conditions (see for example, [36], [41], [42]). Following [37], [38] a model for such a scenario would almost certainly needed changing a fluid model to a non-Newtonian to connect with observations in actual systems. Alternatively, the nonlinear coupled ordinary differential equations (9)–(11) subject to boundary conditions (12)–(14) can be solved by employing the semi-analytical methods [43], [44]. The stability of the problem understudy could be easily conducted by using the new algorithm presented in [44]. Clearly there is an opportunity for experimental work on these systems.
